# Practical screening of purified cellobiohydrolases and endoglucanases with α-cellulose and specification of hydrodynamics

**DOI:** 10.1186/1754-6834-3-18

**Published:** 2010-08-18

**Authors:** Gernot Jäger, Zhuojun Wu, Kerstin Garschhammer, Philip Engel, Tobias Klement, Roberto Rinaldi, Antje C Spiess, Jochen Büchs

**Affiliations:** 1 AVT-Aachener Verfahrenstechnik, Biochemical Engineering, RWTH Aachen University, Worringerweg 1, D-52074 Aachen, Germany; 2 Max-Planck-Institut für Kohlenforschung, Kaiser-Wilhelm-Platz 1, D-45470 Mülheim an der Ruhr, Germany

## Abstract

**Background:**

It is important to generate biofuels and society must be weaned from its dependency on fossil fuels. In order to produce biofuels, lignocellulose is pretreated and the resulting cellulose is hydrolyzed by cellulases such as cellobiohydrolases (CBH) and endoglucanases (EG). Until now, the biofuel industry has usually applied impractical celluloses to screen for cellulases capable of degrading naturally occurring, insoluble cellulose. This study investigates how these cellulases adsorb and hydrolyze insoluble α-cellulose − considered to be a more practical substrate which mimics the alkaline-pretreated biomass used in biorefineries. Moreover, this study investigates how hydrodynamics affects cellulase adsorption and activity onto α-cellulose.

**Results:**

First, the cellulases CBH I, CBH II, EG I and EG II were purified from *Trichoderma reesei *and CBH I and EG I were utilized in order to study and model the adsorption isotherms (Langmuir) and kinetics (pseudo-first-order). Second, the adsorption kinetics and cellulase activities were studied under different hydrodynamic conditions, including liquid mixing and particle suspension. Third, in order to compare α-cellulose with three typically used celluloses, the exact cellulase activities towards all four substrates were measured.

It was found that, using α-cellulose, the adsorption models fitted to the experimental data and yielded parameters comparable to those for filter paper. Moreover, it was determined that higher shaking frequencies clearly improved the adsorption of cellulases onto α-cellulose and thus bolstered their activity. Complete suspension of α-cellulose particles was the optimal operating condition in order to ensure efficient cellulase adsorption and activity. Finally, all four purified cellulases displayed comparable activities only on insoluble α-cellulose.

**Conclusions:**

α-Cellulose is an excellent substrate to screen for CBHs and EGs. This current investigation shows in detail, for the first time, the adsorption of purified cellulases onto α-cellulose, the effect of hydrodynamics on cellulase adsorption and the correlation between the adsorption and the activity of cellulases at different hydrodynamic conditions. Complete suspension of the substrate has to be ensured in order to optimize the cellulase attack. In the future, screenings should be conducted with α-cellulose so that proper cellulases are selected to best hydrolyze the real alkaline-pretreated biomass used in biorefineries.

## Background

Lignocellulose is a renewable resource that can be used for the sustainable production of platform chemicals or fuels [1,2]. Essential for its industrial use is the hydrolysis of its main component cellulose to glucose involving at least three different types of cellulases [3-6]: cellobiohydrolase (CBH, EC 3.2.1.91), endoglucanase (EG, EC 3.2.1.4) and β-glucosidase (EC 3.2.1.21). As the cellulose depolymerization performed by CBHs and EGs is the rate-limiting step for the whole hydrolysis [7], screening for CBHs and EGs is important. However, CBHs and EGs are often characterized with different impractical model substrates that do not mimic the real biomass in biorefineries [7]. Thus, screening experiments need to be conducted with a more practical substrate such as α-cellulose so that proper cellulases are selected which best hydrolyze the biomass actually used in biorefineries.

**Figure 1:**
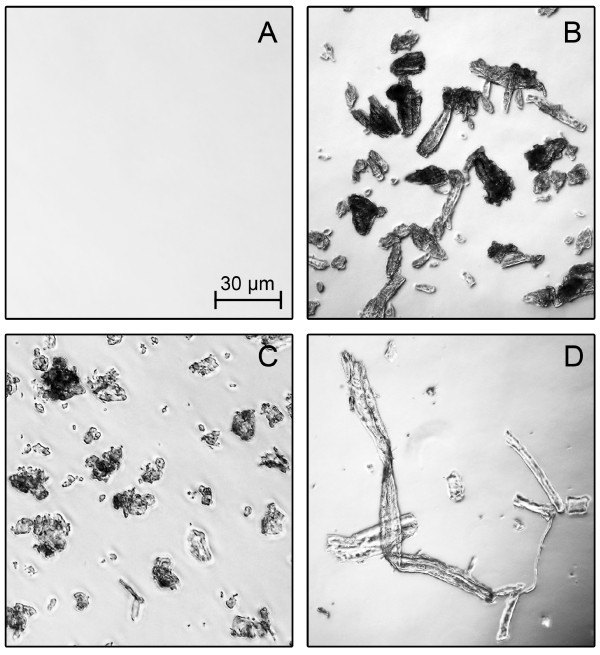
**Light microscopic pictures of the applied cellulosic substrates**. (A) Carboxymethyl cellulose; (B) Avicel PH101; (C) Sigmacell 101; (D) α-cellulose. 10 g/L cellulose in 0.1 M sodium acetate buffer at pH 4.8. Eclipse E600 (Nikon, Tokyo, Japan).

α-Cellulose is a solid residue of lignocellulose after extraction with strong alkali [8-10] and mainly consists of cellulose and a small amount of hemicellulose (Table 1) [11]. α-Cellulose exhibits similar crystallinity and porosity to wood biomass [12] and shows the natural structure of cellulose fibres (Figure 1). Up to now, it has just been used for assaying total cellulase activity [7]. In contrast, conventional model substrates, further processed from α-cellulose, are more artificial [8], because they are dyed, derivatized or water-soluble and show unnatural physical properties (such as crystallinity, degree of polymerization, porosity) [7]. Consequently, α-cellulose is more natural and most similar to alkaline-pretreated cellulosic biomass used in biorefineries [7].

**Table 1:** Physical properties and product information of applied cellulosic substrates

Substrate	Solubility in water	Impurities	*CrI *[%]	*DP_w_* [AGU]	*d_p_* [μm]	Brand	Product code
CMC	Soluble*	Pure*	-	400^‡^	-	Fluka^§^	21900
Avicel PH101	Insoluble*	Pure*	82	200 - 240	43.82	Fluka^§^	11635
Sigmacell 101	Insoluble*	Pure*	Amorphous^¶^	1590 - 1960	15.86	Sigma^§^	S6790
α-Cellulose	Insoluble*	impure: Xylan^†^	64	2140 - 2420	68.77	Sigma^§^	C8002

* Reference [7].

^† ^Reference [11].

^‡ ^According to manufacture's data.

^§ ^Fluka and Sigma are subsidiaries of Sigma-Aldrich.

^¶ ^In the case of Sigmacell 101, no clearly resolved X-ray diffraction profiles were detected. Instead, a smooth peak was detected which is typical for amorphous cellulose according to Hall *et al*. [107]. Pala *et al*. [37] found similar results for Sigmacell 101.

*CrI*, the crystallinity index of cellulose; *DP_w_*, the weight-average degree of polymerization;

*d_P_*, the geometric mean particle size; CMC, carboxymethyl cellulose.

Since α-cellulose is insoluble, the adsorption of cellulases onto α-cellulose is a prerequisite for hydrolysis [4,12,13]. Cellulase adsorption is usually analysed using the Langmuir isotherm [14]. It assumes a single, reversible adsorption step to uniform cellulose binding sites without interactions among cellulases. However, according to various authors, the cellulase adsorption onto the respective cellulose was found to be irreversible [15-17]. In addition, cellulase interactions [18,19], cellulose heterogeneity and porosity were also cited [20-22]. Consequently, several alternative adsorption models were developed [23-26]. Nevertheless, the Langmuir isotherm is the most common mechanistic model for cellulase adsorption [4,12,27,28] and is easily interpretable. Besides the applied cellulases and substrates, temperature is especially important as it affects cellulase adsorption. The amount of adsorbed cellulase is decreased with increasing temperature [16,29-31].

Few cellulase adsorption studies have been performed using α-cellulose or other fibrous substrates [14], and these studies utilized complex cellulase systems [32-35]; as yet, no purified cellulases have been analysed. As insoluble substrates are applied, attention has to be paid to hydrodynamics. Until now, cellulase adsorption and activity have not been investigated systematically by considering liquid mixing and particle suspension.

In this study, insoluble α-cellulose is proposed as a more practical substrate to screen for purified CBHs and EGs. Moreover, this study investigates and correlates in detail cellulase adsorption and activity under different hydrodynamic conditions.

## Methods

### Cellulosic substrates

The cellulosic substrates carboxymethyl cellulose (CMC), Avicel PH101, Sigmacell 101 and α-cellulose (Figure 1) were purchased from Sigma-Aldrich (MO, USA). The physical properties and product information are presented in Table 1. The crystallinity index (*CrI*) was determined by powder X-ray diffraction (XRD) (STOE & Cie GmbH, Darmstadt, Germany). XRD patterns were obtained using Cu*Kα *radiation, a diffraction angle 2*θ *from 10° to 30° (step size of 0.02°) and a counting time of 2 s. The *CrI *was calculated using the equation *CrI *[%] = (*I_002_*-*I_AM_*)/*I_002 _* × 100, where *I_002_* is the maximum intensity of the crystalline plane (002) reflection (2*θ = *22.5°) and *I_AM_* is the intensity of the scattering for the amorphous component at about 18° in cellulose-I [36]. Different *CrI *values for Sigmacell 101 can be found in the literature [37,38]. This may be explained by the varying quality of cellulose depending on batches and production location [39]. Nevertheless, Sigmacell 101 is typically chosen as a more amorphous cellulose [38]. The weight-average degree of polymerization (*DP_w_*) was determined by gel permeation chromatography [40]. The geometric mean particle size (*d_P_*) was analysed by laser diffraction [41] using a LS13320 (Beckman Coulter, CA, USA).

### Purification of cellulases

The commercial cellulase preparation Celluclast^® ^1.5L (Novozymes, Bagsværd, Denmark) was applied to purify the cellulases CBH I, CBH II, EG I and EG II by using column chromatography, with an Äkta FPLC (GE Healthcare, Buckinghamshire, UK) which automatically measures conductivity and ultraviolet absorbance at 280 nm. All the columns were purchased from GE Healthcare. In addition, chromatographic experiments were carried out at room temperature and the automatically collected fractions were directly cooled at 4°C. For anion exchange chromatography, 7.5 mL Celluclast^® ^was previously rebuffered using 0.05 M Tris-HCl buffer (pH 7) and Sephadex G-25 Fine (dimensions: 2.6 cm × 10 cm) at 110 cm/h. The rebuffered sample was loaded on DEAE-Sepharose (dimensions: 1.6 cm × 10 cm) at 60 cm/h using 0.05 M Tris-HCl (pH 7) as a running buffer. The bound proteins were eluted stepwise (35% v/v, 100% v/v) with 0.2 M sodium chloride in 0.05 M Tris-HCl buffer (pH 7). Furthermore, hydrophobic interaction chromatography was performed with 1 M ammonium acetate buffer (pH 5.5) and phenyl-Sepharose (dimensions: 1.6 cm × 2.5 cm) at 30 cm/h. No additional salts had to be added as ammonium effectively promotes ligand-protein interactions in hydrophobic interaction chromatography [42,43]. After loading of a rebuffered sample, the bound proteins were eluted with 0.05 M ammonium acetate buffer (pH 5.5). Moreover, cation exchange chromatography was performed with 0.02 M sodium acetate buffer (pH 3.6) and SP-Sepharose (dimensions: 1.6 cm × 2.5 cm) at 60 cm/h. The rebuffered sample was loaded and bound proteins were eluted stepwise (15% v/v, 100% v/v) with 1 M sodium chloride in 0.02 M sodium acetate buffer (pH 3.6). Finally, when size exclusion chromatography (SEC; dimensions: 1.6 cm × 60 cm) was applied, a 0.6 mL sample was directly injected using 0.01 M sodium acetate buffer (pH 4.8) and Sephacryl S-200 HR at 15 cm/h.

### Measurement of protein concentration

After cellulase purification, the protein concentrations of the final samples were analysed by the bicinchoninic acid assay [44] using the BCA Protein Assay Kit (Thermo Fisher Scientific, MA, USA) and bovine serum albumin as a standard. The absorbance at 562 nm was measured with a Synergy 4 microtitre plate reader (BioTek Inst, VT, USA).

### SDS-polyacrylamide gel electrophoresis

SDS-polyacrylamide gel electrophoresis [45] was applied to analyse the identity and purity of single cellulases. Novex^® ^12% polyacrylamide Tris-Glycine gels (Invitrogen, CA, USA) and samples were prepared according to the manufacturer's protocol. A prestained protein marker (New England Biolabs, MA, USA) was used as a molecular mass marker. Finally, the proteins were stained with coomassie brilliant blue [46] and analysed densitometrically using the scanner Perfection V700 (Epson, Suwa, Japan).

### Adsorption experiments

Adsorption experiments were performed in 0.1 M sodium acetate buffer (pH 4.8) using 10 g/L α-cellulose and various amounts (see below) of the particular cellulases CBH I and EG I. Solutions with α-cellulose and solutions with cellulases were preincubated separately at 45°C for 10 min and experiments were started by mixing both solutions. The final mixtures were incubated as duplicates in 2 mL Eppendorf tubes with a filling volume *V_L_* = 1 mL on a thermo mixer MHR23 (HLC Biotech, Bovenden, Germany) with a shaking diameter *d_0_* = 3 mm. Blanks, either without cellulase, neither substrate nor cellulase or without substrate were incubated similarly. The reaction was stopped by centrifugation (8000 g, 1 min), and the supernatants were immediately analysed for unbound cellulase using the bicinchoninic acid assay. As single cellulases and short incubation times were applied, only small amounts of reducing sugars were produced and, therefore, cellulase adsorption could be analysed by the bicinchoninic acid assay [47]. The adsorbed cellulase concentration was calculated as the difference between initial (blanks) and unbound cellulase concentration.

In order to determine adsorption isotherms, the cellulase concentrations were varied between 0.01 g/L and 1.25 g/L. Preliminary adsorption kinetics showed that an incubation time of 40 min was needed to reach equilibrium. The shaking frequency was *n *= 1000 rpm to exclude mass transfer limitations. Adsorption parameters were determined using the Langmuir isotherm [14]:

(1) $$A=\frac{A_{\max}\cdot E}{K_{D}+E}$$

in which: *A *denotes the amount of adsorbed cellulase (μmol_cellulase_/g_cellulose_); *A_max_*, the maximum cellulase adsorption at equilibrium (μmol_cellulase_/g_cellulose_); *E*, the free cellulase concentration (μmol_cellulase_/L); and *K_D_*, the dissociation constant (μmol_cellulase_/L).

In order to determine adsorption kinetics, the final cellulase concentration was 0.9 g/L, and the incubation time was varied between 0-100 min. Different shaking frequencies *n *= 0-1000 rpm were chosen to analyse hydrodynamic effects. Parameters for adsorption kinetics were determined using simple pseudo-first-order kinetics [48]:

(2) $$A(t)=A_{\max}\cdot (1-e^{\left(-k_{ad}\cdot t\right)})$$

in which *A(t) *is the amount of adsorbed cellulase (μmol_cellulase_/g_cellulose_) at time *t *(s), and *k_ad_* reflects the pseudo-first-order adsorption rate constant for approaching equilibrium (s^-1^).

### Activity experiments and sugar analysis

Cellulase activity assays with a final concentration of 10 g/L cellulose and 0.1 g/L enzyme were performed in 0.1 M sodium acetate buffer (pH 4.8). The mixtures were incubated as triplicates in 2 mL Eppendorf tubes with *V_L _* = 1 mL on a thermo mixer MHR23 at 45°C, *n *= 0-1000 rpm and *d_0_* = 3 mm. Depending on the substrate applied, the incubation time was: 10 min for CMC; 120 min for Avicel PH101; 30 min for Sigmacell 101; and 60 min for α-cellulose. The cellulase activities on Avicel and CMC were used to differentiate CBHs and EGs, respectively [49]. Blanks, either without cellulase - neither substrate nor cellulase - or without substrate, were incubated similarly. Preliminary kinetic experiments showed that inhibiting product concentrations were not reached during the applied incubation times. In addition, low enzyme concentrations were applied, so jamming of cellulases could be neglected [50]. After incubation, the reaction was stopped by boiling for 10 min. The amount of released reducing sugars was determined with the dinitrosalicylic acid assay [51]. For accurate determination of low reducing sugar concentrations, 1.25 g/L glucose was added to each sample [52]. The absorbencies were measured at 540 nm in a Synergy 4 microtitre plate reader. Product concentrations were calculated using glucose as a standard and activities were expressed as the unit U (μmol_glucose equivalents_/min). As CBHs and EGs show different product profiles [53], cellulase activities may be underestimated when glucose is used as a standard in reducing sugar assays [7]. Nevertheless, glucose is often applied [49] when analyzing relative changes in single cellulase activities.

### Determination of hydrodynamics

In order to determine the hydrodynamics during the various adsorption and activity experiments, pictures of the liquid phase with immersed α-cellulose particles were taken at different shaking frequencies. A mixture of 10 g/L α-cellulose in 0.1 M sodium acetate buffer (pH 4.8) was shaken in a transparent 2 mL Eppendorf tube with an inner tube diameter *D_t_* = 1 cm on an orbital shaking platform. The filling volume *V_L_* and the shaking diameter *d_0_* were constant at *V_L_* = 1 mL and *d_0 _* = 3 mm. A miniature charged-coupled device camera XC-777AP (Sony, Tokyo, Japan) was installed on the orbital shaking platform close to the Eppendorf tube, and video images were recorded. At all shaking frequencies, a pause of 5 min was given to allow the suspension to stabilize itself before the next shaking frequency was set. The critical shaking frequency (*n_crit_*) for liquid mixing, depending on the geometric (*d_0_*, *D_t_*, *V_L_*) and physical parameters (liquid density *ρ_L_*, surface tension *σ*) of the applied reaction system, was calculated according to Hermann *et al*. [54]:

(3) $$n_{crit}=\sqrt{\frac{\sigma \cdot D_{t}}{4\cdot \pi \cdot V_{L}\cdot \rho_{L}\cdot d_{0}}}$$

The critical shaking frequency (*n_crit_*) is reached when the labour delivered by the centrifugal force is equal to the surface tension of the liquid. Since sodium acetate is capillary-inactive and cellulose loading was low, both their impacts were negligible.

### Computational methods

After SDS-polyacrylamide gel electrophoresis, the molecular mass and purity of each cellulase were analysed using the software TotalLab TL100 (Nonlinear Dynamics, Newcastle, UK). Parameters of the mathematical adsorption models were calculated by nonlinear, least squares regression analysis using MATLAB version R2008b (The MathWorks, MA, USA).

## Results and discussion

### Purification of cellulases

**Figure 2:**
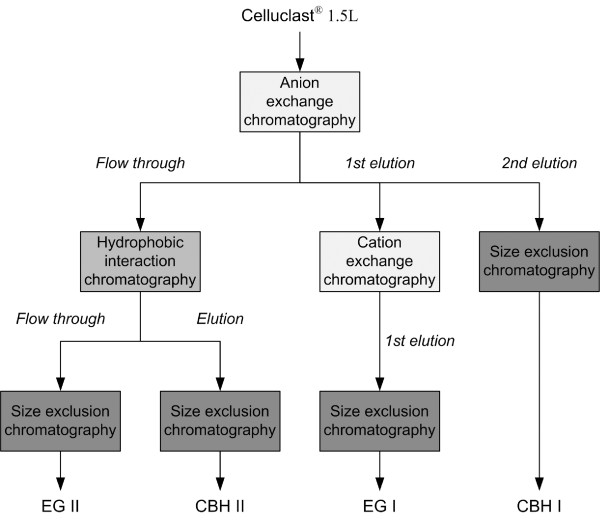
**Flow diagram for the applied chromatographic purification of the individual cellulases**.

The enzyme mixture Celluclast^®^, consisting of cellulolytic and xylanolytic enzymes produced by *T. reesei *[42,55,56], was used as the source material to purify the individual cellulases. According to their relative protein amount the main enzymes are CBH I, CBH II, EG I and EG II [57]. Figure 2 is a flow diagram for the applied chromatographic purification of these various cellulases. After every purification step, the fractions were analysed by SDS-polyacrylamide gel electrophoresis and cellulase activity assays using Avicel and CMC to differentiate CBHs and EGs, respectively (data not shown).

**Figure 3:**
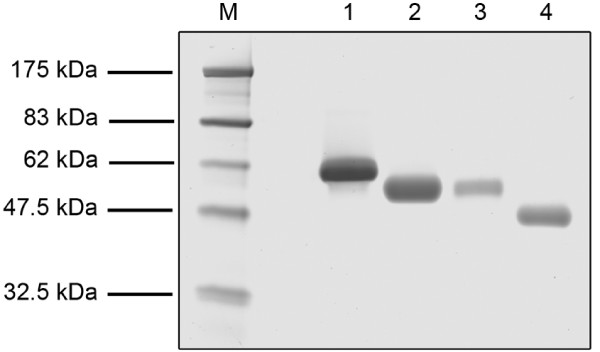
**SDS-polyacrylamide gel electrophoresis of the purified cellulases**. (M) molecular mass marker, (1) cellobiohydrolase (CBH) I, (2) CBH II, (3) endoglucanase (EG) I, (4) EG II. 12% polyacrylamide gel, the same volume of the purified cellulase samples (15 μL) was loaded onto the particular slots.

In order to determine the molecular mass and purity of the individual cellulases, SDS-polyacrylamide gel electrophoresis (Figure 3) and a densitometric analysis [58] were applied. By comparing the molecular masses with the literature [42,55,56,59-62], the following cellulases were identified: CBH I (61 kDa), CBH II (54 kDa), EG I (55 kDa) and EG II (46 kDa). In addition, the amino acid sequence of each cellulase was determined by mass spectrometry [63] and cellulase identities were checked using the Mascot database [64] (data not shown). The final fractions showed cellulases with high purity (≥ 0.99), which is better or comparable with the results of other researchers [42,60,65,66]. In other studies, only two or three of the major *T. reesei *cellulases could be purified [59,60,65] or more purification steps were necessary [55].

### Adsorption isotherms

**Figure 4:**
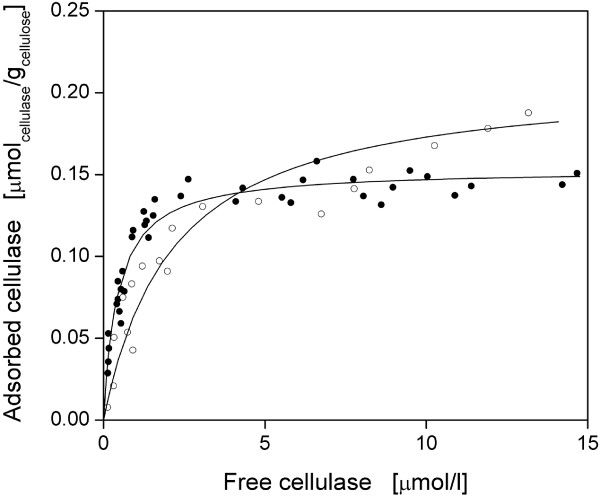
**Adsorption isotherms of the purified cellulases onto α-cellulose**. (Black circle) Cellobiohydrolase (CBH) I; (white circle) endoglucanase (EG) I. Predicted Langmuir isotherms, according to Eqn (1), are shown as solid lines and corresponding parameters are listed in Table 2. 10 g/L α-cellulose in 0.1 M sodium acetate buffer at pH 4.8, *T *= 45°C, *V_L_* = 1 mL, *n *= 1000 rpm, *d_0_* = 3 mm, reaction time 40 min.

As the adsorption of cellulases is a prerequisite for cellulose hydrolysis, adsorption studies were performed by the example of CBH I and EG I. After these were purified, the adsorption isotherms with α-cellulose as a practical cellulosic substrate were determined. Preliminary adsorption kinetics showed that an incubation of 40 min was needed to reach equilibrium. As seen in Figure 4, isotherms of CBH I and EG I showed the adsorption to be a characteristic function of free cellulase concentration. After a sharp increase in adsorbed cellulase at low cellulase concentrations, a plateau was reached at higher concentrations (> 10 μmol/L). In addition, denatured CBH I and EG I, boiled for 10 min, showed no adsorption (data not shown). Therefore, adsorption was specific and required functional protein structures.

In this investigation, the Langmuir isotherm [Eqn (1)] provided a good fit (Figure 4; Table 2). The dissociation constant (*K_D_*), as a reciprocal value for adsorption affinity, was lower for CBH I than for EG I. Different values for *K_D_* have to be derived from differences in cellulose binding modules or catalytic domains. According to Linder *et al*. [67], the cellulose binding modules of CBH I and EG I show single amino acid substitutions leading to differences in binding affinity. In addition, catalytic domains of cellulases are known to specifically adsorb to cellulose binding sites independently of cellulose binding modules [4]. The maximum cellulase adsorption at equilibrium (*A_max_*) was higher for EG I than for CBH I, indicating more accessible cellulose binding sites for EG I, which was also observed by other researchers [19,68]. Besides the aforementioned differences in cellulase structure and binding affinity, these maximum adsorption differences could be explained by the lower molecular mass of EG I and, therefore, a better access to internal binding sites as described for other proteins and materials [69,70]. Nidetzky *et al*. [68] found similar values of *K_D_* and *A_max_* using filter paper as cellulosic substrate (CBH I: 0.71 μmol/L, 0.17 μmol/g; EG I: 1.79 μmol/L, 0.17 μmol/g). Filter paper shows similar *CrI *and *DP_w_* values as α-cellulose [7,12,71] and is used for the measurement of total cellulase activity [49,52]. However, the filter paper assay requires considerable effort and is error-prone [72,73]. Furthermore, many adsorption studies were performed at low temperatures (2-5°C) [24,74-77] to prevent cellulose hydrolysis and thus cellulase desorption [78]. In this study, a more practical temperature of 45°C was selected similar to those temperatures in cellulose hydrolysis. Here, no decrease in adsorbed cellulase was observed.

**Table 2:** Langmuir and kinetic adsorption parameters of purified cellulases using α-cellulose at *n *= 1000 rpm

Cellulase	Langmuir adsorption parameters*			Kinetic adsorption parameters^†^		
	*A_max_* [μmol/g]	*K_D_* [μmol/L]	*R² *[-]	*A_max_* [μmol/g]	*k_ad_* [s^-1^]	*R² *[-]
CBH I	0.155^‡ ^± 0.003	0.433 ± 0.039	0.93	0.170^‡ ^± 0.003	0.0031 ± 0.0002	0.98
EG I	0.212 ± 0.010	2.146 ± 0.216	0.90	0.213 ± 0.007	0.0019 ± 0.0002	0.96

* According to Eqn (1).

^† ^According to Eqn (2).

^‡ ^Differences of *A_max_* resulted because different charges of CBH I were used.

Errors are given as standard deviations.

*A_max_*, the maximum cellulase adsorption at equilibrium; *K_D_*, the dissociation constant; *k_ad_*, the pseudo-first-order adsorption rate constant; CBH, cellobiohydrolase; EG, endoglucanase.

### Adsorption kinetics

**Figure 5:**
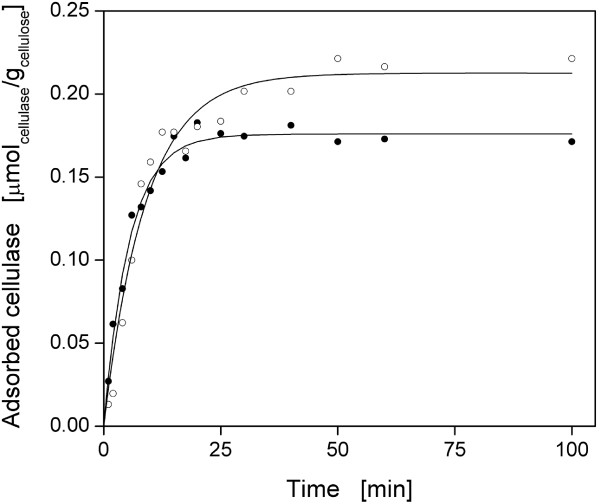
**Adsorption kinetics of the purified cellulases onto α-cellulose**. (Black circle) cellobiohydrolase (CBH) I; (white circle) endoglucanase (EG) I. Predicted adsorption kinetics, according to Eqn (2), are shown as solid lines and corresponding parameters are listed in Table 2. 10 g/L α-cellulose in 0.1 M sodium acetate buffer at pH 4.8, *T *= 45°C, *V_L_* = 1 mL, *n *= 1000 rpm, *d_0_* = 3 mm, 0.9 g/L cellulase.

According to Figure 5, the adsorption kinetics of CBH I and EG I on α-cellulose were determined. For both cellulases, the respective adsorption rose quickly until a final plateau was reached. The final amount of adsorbed cellulase did not change with further incubation. Seemingly, α-cellulose does not contain many micropores [79] that can only be penetrated slowly by cellulases [80]. As shown in Table 2, the simple pseudo-first-order kinetic model [Eqn (2)] provided a good fit. Taking experimental errors into account, similar values for *A_max_* were determined as in adsorption isotherm experiments and, thus, complete saturation of α-cellulose was reached in kinetic studies. The kinetic constant (*k_ad_*) was higher for CBH I than for EG I and thus saturation was reached after 25 min and 40 min, respectively. As with filter paper, similar incubation times were found for CBH I and EG I [68]. Cellulase adsorption is rapid compared to the time required for complete hydrolysis [4,12]. Depending on the applied cellulose, the adsorption equilibrium is normally reached after 30-90 min [28,53,80-84].

### Influence of hydrodynamics

**Figure 6:**
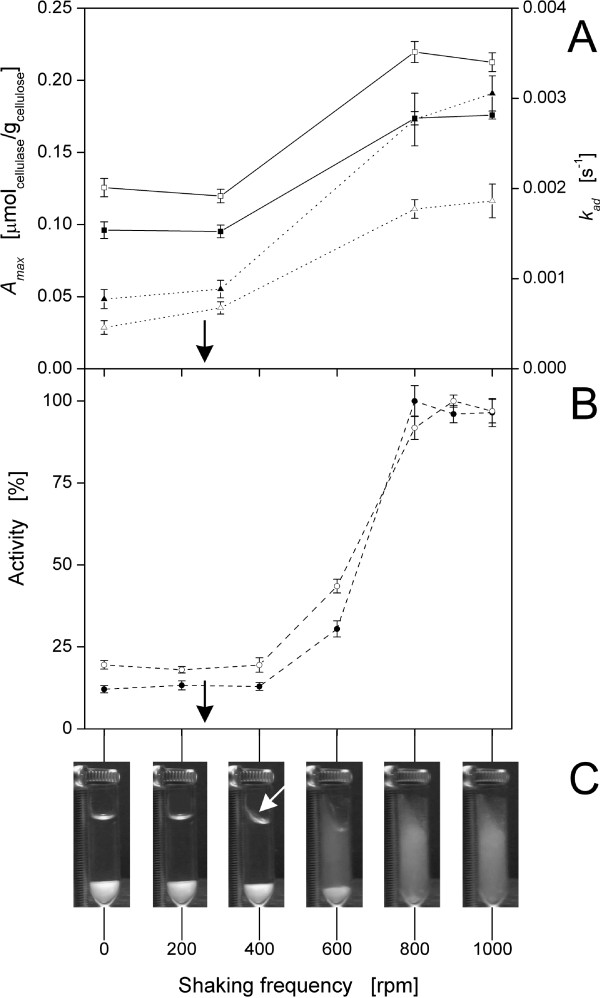
**Adsorption kinetics and activities of the purified cellulases onto α-cellulose at different hydrodynamic conditions**. (A) Influence of shaking frequency on kinetic adsorption parameters (including standard deviations), maximum cellulase adsorption at equilibrium (*A_max_*; solid line) and pseudo-first-order adsorption rate constant (*k_ad_*; dotted line), according to Eqn (2): (black square) *A_max_* of cellobiohydrolase (CBH) I; (white square) *A_max_* of endoglucanase (EG) I, (black triangle) *k_ad_* of CBH I; (white triangle) *k_ad _*of EG I. The critical shaking frequency (*n_crit_*), calculated according to Eqn (3) [54], is indicated with black arrows; (B) Influence of shaking frequency on the activity of cellulases (dashed line): (black circle) CBH I; (white circle) EG I. Relative values (including standard deviations) are standardized to maximum activities (CBH I: 0.25 U/mg; EG I: 0.64 U/mg); (C) pictures of the liquid phase with immersed α-cellulose particles at different shaking frequencies. The white arrow indicates the start of liquid mixing. Images were obtained with a charged-coupled device camera installed on a shaking platform. 10 g/L α-cellulose in 0.1 M sodium acetate buffer at pH 4.8, *T *= 45°C, *V_L_* = 1 mL, *n *= 0-1000 rpm, *d_0 _*= 3 mm.

As α-cellulose is an insoluble substrate, the hydrodynamics of the reaction system have to be taken into account. Therefore, the impact of shaking frequency and the resulting hydrodynamics on the adsorption as well as on the activity of the cellulases were investigated in detail for the first time. Adsorption kinetics of CBH I and EG I were determined for different shaking frequencies using simple pseudo-first-order kinetics (Figure 6A). This model provided a good fit at all shaking frequencies; no biphasic adsorption kinetics with two different adsorption rates were observed [68]. At all applied shaking frequencies and as seen in prior experiments (Table 2), *A_max_* (solid line) was higher for EG I and *k_ad_* (dotted line) was higher for CBH I. Between 0 rpm and 300 rpm, *A_max_* and *k_ad_* were almost constant for both cellulases, whereas for both a sharp increase could be determined between 300 rpm and 800 rpm. Above 800 rpm, only a slight increase in *k_ad_* was observed. Consequently, enhanced mixing improved the contact between cellulase and substrate [85], and, therefore, the mass transfer and the kinetic constant *k_ad_* increased. However, also the maximum cellulase adsorption *A_max_* rose with enhanced mixing, which was not observed for CBH I and EG I using filter paper as a cellulosic substrate [68]. This may be explained by a better exposure of α-cellulose to the liquid and, therefore, a better cellulose surface accessibility for cellulase adsorption.

As seen in Figure 6B, the activities of CBH I and EG I were also investigated at different shaking frequencies using α-cellulose as substrate. For both cellulases, the same trend in activity was observed, whereby a sharp increase occurred between 400 rpm and 800 rpm as in the adsorption kinetic experiments (Figure 6A). Consequently, higher shaking frequencies clearly improved the adsorption of cellulases, thereby bolstering their respective activity, because adsorption is a prerequisite for cellulose hydrolysis [4,12,13]. Thus, when short incubation times of cellulases are applied (for example, washing agent), catalyst optimization should also be focused on improving the cellulase binding properties. In other studies, the effect of agitation on cellulose hydrolysis was investigated without considering adsorption. These studies showed that enhanced agitation increases initial cellulose hydrolysis rates [85-87]. However, attention has to be paid to cellulase inactivation reducing the final yield of cellulose hydrolysis [88]. This is especially important when using high solid concentrations [89] and shear-force sensitive cellulases [90]. In this current study, however, low solid concentrations, short incubation times and a shaken system were applied, so cellulase inactivation could be neglected. Moreover, upon using immobilized or displayed cellulases [91-93], lower shaking frequencies are beneficial to ensure sufficient surface contact between cellulase and solid substrate [94]. In comparison to the adsorption parameters, a disproportionate increase in cellulase activity was observed with enhanced agitation (Figure 6A and B). As CBHs and EGs are inhibited by soluble hydrolysis products, such as glucose and cellobiose [95-97], agitation may transport these inhibiting products away from the cellulases, thus decreasing the local concentration of inhibiting products and improving the cellulase activity.

In order to understand the influence of shaking frequency on the adsorption and cellulase activity, the hydrodynamics inside the respective reaction tube were investigated in detail. Pictures of the liquid phase with immersed α-cellulose particles were taken at different shaking frequencies (Figure 6C). For *n *≤ 200 rpm, the liquid surface remained horizontal and no liquid mixing was observed. Once *n *≥ 400 rpm, liquid mixing started (white arrow; Figure 6C). According to Hermann *et al*. [54], a critical shaking frequency (*n_crit_*) is necessary for liquid mixing and can be calculated according to Eqn (3). In this current investigation, *n_crit_* was 260 rpm (black arrows; Figure 6A and B), which fitted well to the start of liquid mixing between 200 rpm and 400 rpm. The boundary layer at the cellulose-liquid interface is relatively thick without mixing [98] and can decrease the rate of cellulase adsorption. However, mixing was increased by exceeding *n_crit_*, leading to a decrease in the width of the boundary layer at the cellulose-liquid interface. Since adsorption kinetics and cellulase activity did not significantly change between 0 rpm and 400 rpm, liquid mixing was not the rate limiting step.

A sharp increase in adsorption parameter values and cellulase activities was observed once suspension of cellulose particles began (*n *= 600 rpm). As soon as the particles were completely suspended (*n *= 800 rpm), their whole surface was exposed to the liquid and all external cellulose binding sites were accessible to the cellulases. Hence, an optimal particle-liquid mass transfer was achieved [99] and the parameters *A_max_*, *k_ad_* as well as cellulase activities reached their maximum values. Complete suspension is defined as the point when no particles are deposited on the tank bottom for longer than one second [100]. This criterion is designated as the just suspending speed or off bottom speed. A correlation for calculating the just suspending speed in shaking vessels can be found in the literature [101]. However, it can not be applied to cellulose particles because of their fibrous structure and wide particle size distribution. Complete suspension is known to be required for high cellulase activity [102]. However, this current paper shows for the first time the effect of suspension on cellulase adsorption as well as the correlation between the adsorption and the activity of cellulases at different hydrodynamic conditions.

### Cellulase activity with conventional model substrates and α-cellulose

**Figure 7:**
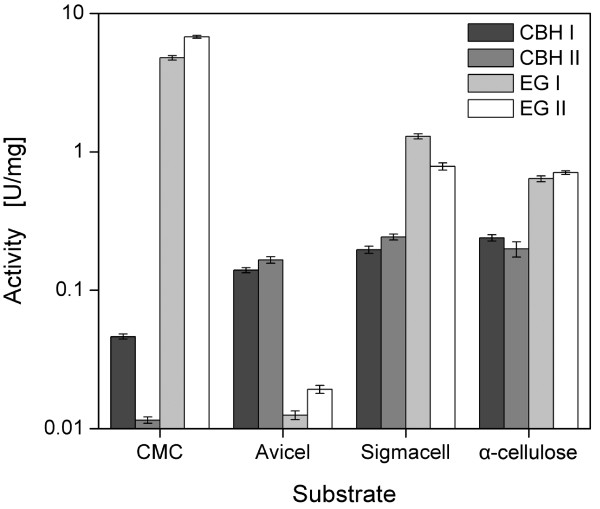
**Activities of the purified cellulases onto different cellulosic substrates**. 10 g/L cellulose in 0.1 M sodium acetate buffer at pH 4.8, *T *= 45°C, *V_L_* = 1 mL, *n *= 1000 rpm, *d_0_* = 3 mm, 0.1 g/L cellulase.

After the adsorption and activity of purified cellulases at various hydrodynamic conditions were studied with α-cellulose, the cellulase activities on different artificial model substrates and on α-cellulose were finally compared (Figure 7). EG I and EG II showed high specific activities towards CMC and low activities towards Avicel. Regarding CBH I and CBH II, the opposite was observed. Since Avicel and CMC are model substrates to differentiate CBHs and EGs, these results are in good agreement with the literature [7,49,103,104]. However, when comparing CBHs and EGs, a common cellulosic substrate is necessary. In the case of Sigmacell, the activities of EGs were just six-times higher than the activities of CBHs. As Sigmacell is an insoluble, unsubstituted cellulose with low *CrI*, it can be hydrolyzed by CBHs and EGs [4]. Sigmacell, however, is an artificial substrate processed from α-cellulose [7,8] and does not mirror the actual biomass present in a biorefinery. By using insoluble α-cellulose, the activities were very similar and the ratio between EGs and CBHs was approximately 2.7:1. α-Cellulose is normally used for total cellulase activity measurements [7,72]. As α-cellulose mimics the alkaline-pretreated biomass used in biorefineries, α-cellulose is suggested as an excellent substrate in early experiments to screen for apt cellulases to process practical cellulosic substrates.

## Conclusions

In this study, insoluble α-cellulose was found to be an excellent practical substrate to characterize and screen for purified CBHs and EGs. First, a novel and reproducible purification method was established to prepare the major cellulases from *T. reesei *with high purity. Second, the adsorption isotherms and kinetics of the purified cellulases were analysed for the first time using α-cellulose as a cellulosic substrate. Here, the calculated adsorption parameters (*A_max_*, *K_D_*, *k_ad_*) of the studied cellulases were comparable to those for filter paper as an established model substrate. In addition, this investigation shows in detail, for the first time, the effect of hydrodynamics on cellulase adsorption as well as the correlation between the adsorption and the activity of cellulases at different hydrodynamic conditions. Complete suspension of α-cellulose particles clearly enhanced the adsorption of cellulases thereby augmenting cellulase activity. By comparing conventional model substrates with α-cellulose, CBHs and EGs showed similar cellulase activities only on insoluble α-cellulose.

Even though other researchers use conventional pure cellulosic substrates, these are not suitable for characterizing purified CBHs and EGs. Instead, α-cellulose is ideal when alkaline pretreatment is considered as a previous pretreatment step. In the future, screening experiments should be conducted with α-cellulose so that proper cellulases are selected to best hydrolyze the real alkaline-pretreated biomass used in biorefineries. In addition, α-cellulose can be used in automated screening platforms [72] as suspensions of α-cellulose particles can be handled by pipetting. Since lignocellulose pretreatment in biorefineries significantly alters the structure of cellulose, cellulases should be characterized with other practical cellulosic substrates that represent other pretreatment techniques [105,106] (such as ammonia fibre explosion, ionic liquid process, organosolv process and steam explosion).

## Abbreviations

CBH: cellobiohydrolase; CMC: carboxymethyl cellulose; EG: endoglucanase; SEC: size exclusion chromatography; XRD: X-ray diffraction; *A*: adsorbed cellulase (μmol_cellulase_/g_cellulose_); *A_max_*: maximum cellulase adsorption at equilibrium (μmol_cellulase_/g_cellulose_); *CrI*: crystallinity index (%); *d_0_*: shaking diameter (mm); *d_p_*: geometric mean particle size (μm); *DP_w_*: weight-average apparent degree of polymerization (AGU); *D_t_*: inner tube diameter (mm); *E*: free cellulase concentration (μmol_cellulase_/L); *I_002_*: maximum intensity of the crystalline plane (002) reflection (s^-1^); *I_AM_*: XRD scattering for the amorphous component at 18° in cellulose-I (s^-1^); *k_ad_*: pseudo-first-order adsorption rate constant (s^-1^); *K_D_*: dissociation constant (μmol_cellulase_/L); *n*: shaking frequency (rpm); *n_crit_*: critical shaking frequency for liquid mixing (rpm); *t*: time (s); *T*: temperature (°C); *V_L_*: filling volume (mL); *θ*: diffraction angle (°); *ρ_L_*: liquid density (kg/L); *σ*: surface tension (N/m)

## Competing interests

The authors declare that they have no competing interests.

## Authors' contributions

GJ designed and carried out experiments, analysed results and wrote the manuscript. ZW carried out the cellulase purification and the binding assays. KG carried out the activity assays. PE and TK participated in the method development. RR carried out the measurements of *CrI *and *DP_w_*. AS reviewed the manuscript. JB coordinated the study and reviewed the manuscript. All authors read and approved the final manuscript.
